# Promoting equity in breastfeeding through peer counseling: the US Breastfeeding Heritage and Pride program

**DOI:** 10.1186/s12939-021-01408-3

**Published:** 2021-05-27

**Authors:** Elizabeth C Rhodes, Grace Damio, Helen Wilde LaPlant, Walter Trymbulak, Carrianne Crummett, Rebecca Surprenant, Rafael Pérez-Escamilla

**Affiliations:** 1 Yale School of Medicine, 333 Cedar Street, 06510 New Haven, Connecticut, USA; 2 Yale School of Public Health, 135 College Street, 06510 New Haven, Connecticut, USA; 3 Hispanic Health Council, 175 Main Street, 06106 Hartford, Connecticut USA; 4 Saint Francis Hospital and Medical Center, Trinity Health Of New England, 114 Woodland Street, 06105 Hartford, Connecticut USA

**Keywords:** Lactation, Breastfeeding, Equity, Peer counseling, United States, Ethnic minority

## Abstract

**Background:**

In the United States, Black and Hispanic mothers have lower breastfeeding rates compared with White mothers. To address breastfeeding inequities, the Breastfeeding Heritage and Pride program (BHP) provides breastfeeding support for predominately low-income minority mothers in Connecticut and Massachusetts. We described the process of designing BHP, the program model, and its impact on breastfeeding outcomes.

**Methods:**

This BHP case study is based on in-depth interviews with BHP designers and implementers, peer counselors, and clients; a literature review of BHP impact evaluation studies; and a review of BHP materials. To guide the analysis and organize results, we used the Community Energy Balance Framework, an equity-oriented, multi-level framework for fostering healthy lifestyles.

**Results:**

The Hispanic Health Council designed BHP to address barriers to breastfeeding identified through formative qualitative research with the Latino community, namely lack of role models, limited social support, embarrassment when breastfeeding in public, lack of breastfeeding knowledge, and a norm of formula feeding. According to the BHP model, clients receive education and support through in-person home and hospital visits supplemented by phone calls, beginning prenatally and continuing through one year postpartum. Counseling is delivered by peer counselors, women who have successfully breastfed, have similar cultural roots and life experiences as the clients they serve, and have completed intensive training on lactation management and communication skills. International Board Certified Lactation Consultants provide clinical guidance and ongoing training to peer counselors, as well as direct support to clients, if more specialized knowledge and clinical expertise is needed. Clients facing housing and food insecurity or other socio-economic obstacles that may negatively influence breastfeeding and health and well-being more broadly are connected to other health and social services needed to address their social determinants of health needs, including health care access and food and rent assistance programs. To continuously improve service delivery, BHP has a robust monitoring and evaluation system. In two randomized-controlled trials, BHP was shown to improve breastfeeding initiation and duration of any and exclusive breastfeeding.

**Conclusions:**

BHP highlights the importance of community-engaged formative research for informing breastfeeding program design. It also provides an evidence-based example of a program model that offers a continuum of breastfeeding support, considers cultural-contextual influences on breastfeeding and social determinants of health, and incorporates continuous quality improvement.

**Table:** 

This article is a part of the Interventions and policy approaches to promote equity in breastfeeding collection, guest-edited by Rafael Pérez-Escamilla, PhD and Mireya Vilar-Compte, PhD	

## Background

Breastfeeding provides numerous health benefits, along with economic savings to families and health care systems [1–3]. For children, breastfeeding improves survival and is protective against infections and obesity [1]. Women who breastfeed have a lower risk of hypertension, type 2 diabetes, and ovarian and breast cancers [2, 4, 5]. The American Academy of Pediatrics recommends that infants receive only breast milk for about the first six months of life, followed by the introduction of complementary food alongside continued breastfeeding until the child is at least one year [6]. Although breastfeeding rates have increased steadily in the United States (US) over the past two decades, large inequities in breastfeeding rates persist [7–9]. For example, low-income women consistently have the lowest rates of exclusive breastfeeding at 6 months and any breastfeeding at 12 months [10]. Among infants born in 2017, 21 % of Black infants and 22 % of Hispanic infants were exclusively breastfeeding through 6 months, compared to nearly 30 % of White infants [11]. Black and Hispanic populations also bear the largest disease burden due to suboptimal breastfeeding practices [3].

Breastfeeding inequities can be addressed through evidence-based interventions to promote, protect, and support breastfeeding among low-income and minority women [12]. Even though there are no differences in prenatal breastfeeding intentions between Black and White women, and a higher percentage of Hispanic women intend to breastfeed [8], Black and Hispanic women are less likely to meet their prenatal breastfeeding intentions than White women [8]. An important factor for this inequity may be that some minority women may not have access to effective breastfeeding support to prevent or address latching problems, breast pain, and difficulties with milk production [6, 7, 13]. Evidence-based interventions to support women in breastfeeding exist [13, 14], and while there has been substantial progress in scaling these interventions [15–17], much work remains to ensure every woman who wishes to breastfeed receives the support she needs to meet her goal. Access to maternity care practices that support breastfeeding, such as limiting the use of breast milk supplements, keeping infants with their mothers in the same room after delivery, and postpartum breastfeeding support, is lower among facilities in areas with larger Black populations [18]. For many low-income minority women, professional support from International Board Certified Lactation Consultants (IBCLCs) is unaffordable [10, 19, 20], and ethnic/racial diversity among IBCLCs is lacking [21].

Breastfeeding peer counseling, the provision of breastfeeding education and support by community health workers known as peer counselors [14], is a proven way to promote and support breastfeeding among low-income minority women [19]. Peer counselors are women who have successfully breastfed and have been trained to support other women in their communities to breastfeed [14, 19]. Evidence from randomized-controlled trials has demonstrated that breastfeeding peer counseling improves rates of breastfeeding initiation, duration, and exclusivity [14, 22–24]. Hence, the Department of Health and Human Services has highlighted breastfeeding peer counseling as a key strategy for improving breastfeeding outcomes [19].

The Breastfeeding Heritage and Pride program (BHP) provides breastfeeding peer counseling to over 1,400 predominately low-income minority women in Connecticut and Massachusetts annually. It has been endorsed as an evidence-based program by the Centers for Disease Control and Prevention and National Academies of Science, Engineering and Medicine [25, 26]. In this paper, we describe the process of designing BHP, present the program model for addressing breastfeeding inequities, and summarize the impact of the program on breastfeeding outcomes. This case study offers key learnings for breastfeeding peer counseling programs targeting socio-economically disadvantaged women in the US.

## Methods

This case study draws from three sources of information. First, we used data from in-depth interviews with BHP stakeholders. To conduct the qualitative research, the qualitative research lead (ECR) worked closely with two female research assistants with master’s degrees in public health, one of whom was bi-lingual in English and Spanish. Research assistants underwent a 2-day training covering topics such as best practices for interviewing, the interview guides, and ethics and then received ongoing training and supportive supervision throughout data collection. We used purposive sampling to recruit individuals involved in designing and/or implementing the program in clinical and community settings (BHP designers and implementers) and all current peer counselors, inviting them to participate via email and phone. We also sampled mothers participating in the program (BHP clients) from program enrollment lists using a maximum variation approach to achieve diversity based on the clinic where they receive maternity care, assigned peer counselor, and race/ethnicity. We contacted BHP clients via phone and text message and invited them to participate, sending up to three reminders.

 We conducted interviews in-person and via Zoom Video and Phone between March and August 2020 using semi-structured interview guides tailored to each stakeholder group. The guide used for interviews with BHP clients was translated from English into Spanish. All guides were pilot tested and refined as needed. Interviews with BHP designers and implementers and peer counselors lasted approximately 45 to 60 minutes. Interviews with BHP clients were designed to be shorter to avoid overburdening them and lasted approximately 30 to 45 minutes. We conducted interviews with clients in English or Spanish, according to their preference. Peer counselors and clients received a $30 gift card to compensate them for their time. During all interviews, we used open-ended questions and probing to explore issues in depth. We also used an iterative process of data collection, meaning that as interviews were completed, questions and probes were refined for subsequent interviews to gain more depth and detail on each emerging topic [27].

All interviews were audio-recorded and professionally transcribed verbatim and translated into English, if needed. Each transcript was then checked for accuracy and de-identified to ensure anonymity of participants. A thematic analysis approach was used to analyze the textual data. Codes (issues or topics in the data) were identified and organized into a codebook that included each code name with a code definition and examples of proper use. Textual data were coded using MAXQDA 2020 and coded segments were retrieved for analysis.

Second, we reviewed available BHP materials, such as a formative research report that informed the design of BHP, a training manual for peer counselors [28], operational protocols [29], a community health worker policy brief [30], and reports submitted to program funders. Third, we summarized the results of BHP impact evaluation studies [19, 31–33]. Information from BHP materials and scientific articles helped to triangulate interview findings and provided additional information.

To organize and present the findings, we used the Community Energy Balance (CEB) Framework developed by Kumanyika et al., which depicts considerations for community-based interventions addressing obesity in racial/ethnic minority populations, particularly socially disadvantaged groups [34]. These considerations include types of policies and practices that can promote health at the community level, referred to as intervention targets; settings and change agents for obesity prevention interventions; and cultural-contextual factors that predispose racial/ethnic minority populations to high risk of obesity. The CEB Framework identifies a range of intervention targets, such as food choices and feeding which could be improved through breastfeeding support. It also emphasizes the importance of focusing on multiple intervention settings and agents, including the general population and culture in the host country, racial/ethnic minority communities, households and families, and individuals. BHP focuses on the family and household level by educating women on the benefits of breastfeeding so they can make informed infant feeding choices and supporting women to meet their infant feeding goals. The CEB Framework focuses on diverse cultural-contextual influences, including migration, historical trauma, and racism experienced in different ways by different racial/ethnic minority populations, which aligns with BHP’s goal of addressing these influences on breastfeeding within predominantly racial/ethnic minority communities. We mapped the barriers to breastfeeding BHP seeks to address according to the types of cultural-contextual influences specified by the CEB Framework.

This project was reviewed by the Yale University institutional review board and exempted from requiring human subjects approval.

## Results

We conducted interviews with a total of 42 BHP stakeholders, including 8 program designers and implementers, 6 peer counselors, and 28 BHP clients. Program designers and implementers included individuals from academic institutions, the Hispanic Health Council, and Trinity Health Of New England hospitals in Connecticut and Massachusetts. Peer counselors had an average of 1.7 years of experience delivering BHP services, with a range of 10 months to 3.6 years of experience. Many BHP clients who participated in the study were Black (42.9 %) and/or Hispanic/Latino (42.9 %) and did not have past experience with breastfeeding (60.7 %) (Table 1).

**Table 1:** Characteristics of Breastfeeding Heritage and Pride program clients participating in the study

Characteristics	n (%)
Age, y	
18–21	3 (10.7)
22–34	21 (75.0)
35–44	4 (14.3)
Mean age, y	29
Race	
Black	12 (42.9)
White	7 (25.0)
Bi- or Multi-racial	2 (7.1)
Other	7 (25.0)
Hispanic/Latino	
Yes	12 (42.9)
No	16 (57.1)
Marital Status	
Single	3 (10.7)
With a partner (not married)	10 (35.7)
Married	15 (53.6)
Living with spouse/partner	
Yes	24 (85.7)
No	4 (14.3)
Education	
Some high school	1 (3.6)
High school graduate/general education diploma	10 (35.7)
More than high school	17 (60.7)
Parity	
1	16 (57.1)
> 1	12 (42.9)
Past breastfeeding experience	
Yes	11 (39.3)
No	17 (60.7)

### Development of the program

BHP was established in 1993 by the Hispanic Health Council, a nationally recognized community-based organization promoting the health and social well-being of Latinos and other diverse communities. The Hispanic Health Council was motivated to develop BHP because breastfeeding support in the Latino community in Hartford, Connecticut was severely lacking at the time. Local hospitals provided limited support, and the Special Supplemental Nutrition Program for Women, Infants, and Children (WIC) had not yet launched a breastfeeding peer counseling program. The Hispanic Health Council had also conducted a community-based assessment in Hartford’s Latino community through 31 qualitative interviews with adolescents and women of reproductive age and found that many women faced barriers to breastfeeding initiation and continuation and did not have access to breastfeeding support services.

To design BHP, the Hispanic Health Council conducted formative research to further understand breastfeeding barriers and obtain community input on how to address them. The Hispanic Health Council recruited Latino individuals from programs delivered by local community organizations and conducted four focus groups (two with pregnant and female teen parents, one with young male adults, one with adult parents). Each focus group was facilitated by a trained moderator who used a semi-structured focus group guide and asked follow-up questions based on participants’ responses. A notetaker recorded key issues discussed, and the project team reviewed the notes to identify recurring issues and group them into themes, which were then described in detail in a report.

The results of this formative research highlighted a range of factors that negatively influenced breastfeeding practices in the Latino community (Table 2). Social norms that discouraged breastfeeding were a key sociocultural barrier. Focus group participants thought that some people were uncomfortable with seeing women breastfeed in public spaces. One reason was that breastfeeding was perceived to be related to sexuality, and thus, breastfeeding in public was akin to exposing yourself sexually in public. Some participants acknowledged that they would not feel uncomfortable with breastfeeding if they lived in the past when most women breastfed. Moreover, several participants shared that they had only seen someone breastfeed on television. While most participants shared the view that breast milk is the best source of nutrition for infants, their understanding of the benefits of breastfeeding was limited and, in some instances, inaccurate. They also shared that little or no support for breastfeeding was a significant barrier. Among women who reported breastfeeding, for instance, most shared that they had stopped due to issues such as pain and sore nipples, concern that the baby was not receiving sufficient milk, rejection by the baby, and an unsupportive or misinformed partner. Furthermore, when describing why some women do not breastfeed, participants pointed to lack of information about breastfeeding, difficulty with managing breastfeeding challenges, and lack of role models – for example, not knowing any women who breastfeed. They also described several structural factors impeding breastfeeding, including access to free infant formula (e.g., through government agencies), convenience of infant formula (e.g., bottle feeding as easier than breastfeeding, infant formula as less time-consuming than breastfeeding), and the inability for women to breastfeed when working outside the home.

In interviews, BHP designers called attention to barriers to breastfeeding identified through formative research, as well as additional barriers that they viewed as important, based on their extensive experience working with Latino communities (Table 2). They emphasized the lack of support for breastfeeding within the health care system, pointing out that hospital staff often lack knowledge, experience, or skills to address questions or issues related to breastfeeding. Consistent with the CEB Framework, BHP designers also highlighted the historical experiences and adaptations among Latino communities that negatively influenced breastfeeding, such as breastfeeding no longer being the cultural norm.

**Table 2:** Barriers to breastfeeding mapped to the Community Energy Balance Framework’s cultural-contextual influences

Cultural-contextual influences	Barriers to breastfeeding^a^
Sociocultural influences	• Social norms that discourage breastfeeding • Limited social support for breastfeeding • Lack of role models • Limited knowledge of the benefits of breastfeeding • Lack of self-efficacy to breastfeed • Concept of “las dos cosas” (the belief that providing both breast milk and infant formula benefits infants)
Structural influences	• Disproportionate marketing of infant formula to racial/ethnic minorities • Availability of free infant formula (e.g., through the Special Supplemental Nutrition Program for Women, Infants, and Children) • Difficulty in continuing breastfeeding while working
Historical experiences and adaptations	• Breastfeeding as part of people’s culture was lost over time due to factors such as marketing of infant formula and lack of practices and policies that encourage and support breastfeeding such as adequate maternity leave
Social determinants of health^b^	• Lack of access to health care, including lack of accessible breastfeeding support (e.g., lactation consultants were often unaffordable, clinicians were ill-equipped to provide support, lack of insurance to cover lactation consultants) • Lack of transportation • Housing instability • Food insecurity

^a^Barriers to breastfeeding identified through focus groups with community members as part of formative research and in-depth interviews with BHP designers

^b^The Community Energy Balance Framework recognizes that social determinants of health are the result of cultural-contextual factors [34]

### Program model

BHP is operated by the Hispanic Health Council in partnership with health care organizations, including Trinity Health Of New England and Hartford Hospital. BHP services are provided by peer counselors, who are specialized community health workers hired from the communities BHP serves. While BHP was developed to meet the specific needs of the Latino community in Hartford, Connecticut, the program first became operational within a Hartford Hospital clinic that mainly served Latino patients but also other diverse communities, and therefore, has provided services to all racial/ethnic groups within the patient population since its inception. Currently, the target population of BHP is women giving birth at partnering health care organizations, which serve predominately low-income minority populations in urban and suburban areas in Connecticut and Massachusetts. Although BHP was originally designed to offer services that collectively address cultural-contextual influences on breastfeeding among Latino populations, consistent with the CEB Framework, many of these influences are relevant to other minority and socially disadvantaged populations, and thus the program is applicable for a diversity of women.

**Fig. 1:**
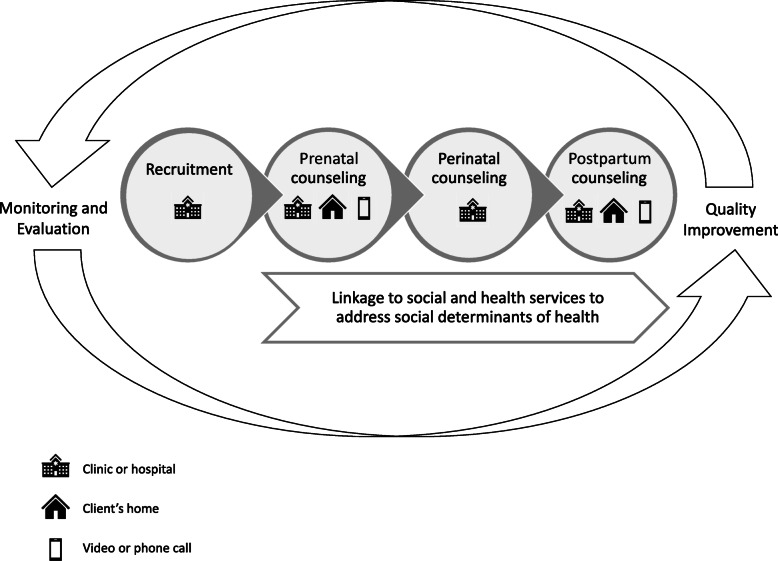
Continuum of breastfeeding support delivered by the Breastfeeding Heritage and Pride program. The Breastfeeding Heritage and Pride program (BHP) offers a continuum of breastfeeding support to clients across time, including the prenatal, perinatal, and postpartum periods, and health care and community settings. Peer counselors provide breastfeeding education and hands-on support with breastfeeding in clinics and hospitals, as well as in clients’ homes. Peer counselors also provide breastfeeding information and support to clients via video calls, phone calls, and text messages. BHP connects clients with other social and health services that can provide assistance with addressing social determinants of health identified by peer counselors that may impede breastfeeding or negatively impact the health and well-being of clients and their families. An integral component of the program is the monitoring and evaluation system, which facilitates continuous improvement of the quality of services

Peer counselors are integrated into prenatal clinics and hospitals operated by and/or affiliated with partnering health care organizations to support the continuum of breastfeeding care (Fig. 1). This integration facilitates enrollment of BHP clients prenatally. Peer counselors either receive referrals from health care providers and then follow up to enroll pregnant women into the program, or directly recruit women in clinics. Once enrolled, BHP assigns each client to a specific peer counselor, allowing the client to develop a trusting relationship with her peer counselor and ensuring continuity of services. Breastfeeding peer counseling begins prenatally and continues for up to one year postpartum. This counseling is provided in-person through home and clinic-based visits that last approximately 30 to 60 minutes, supplemented with video calls, phone calls, and text messages, according to a protocol that specifies the important topics to cover during each visit, as well as the number and timing of visits. Specifically, clients are offered at least: three prenatal visits, which often occur in clinics; one perinatal visit in the hospital immediately after childbirth; and five postpartum home visits, supplemented by seven phone calls. Notably, BHP provides services based on women’s needs and preferences. Some clients opt to not receive all postpartum home visits, if they are breastfeeding successfully and do not need further support, for example. If clients choose to stop breastfeeding, they discontinue visits with peer counselors. Other clients receive additional in-person visits and phone calls, as needed. Peer counselors communicate with clients in English or Spanish based on their preference and provide breastfeeding education and support, such as education on the benefits of breastfeeding, anticipatory guidance to help clients know what to expect ahead of time and prevent challenges to meeting their breastfeeding goals, and hands-on support for resolving breastfeeding challenges. To ensure sufficient capacity for service provision, BHP uses a Community Health Worker Caseload Estimator developed by the Hispanic Health Council in collaboration with a team of national experts to estimate the number of clients each peer counselor can serve per year. The estimation is based on the BHP protocol for services and volume, then modified based on the difference between ideal duration of BHP participation through 12 months postpartum and the reality that for some clients the duration is shorter, as well as real-time observation of peer counselor performance at different caseload volumes [30]. A full-time peer counselor can serve approximately 120 clients per year, with a caseload of approximately 65 clients at any one time.

BHP IBCLCs provide peer counselors with ongoing clinical guidance and training in delivering high-quality breastfeeding counseling to clients. IBCLCs also provide direct support to clients, if more specialized knowledge and clinical expertise is required. A “BHP Peer Counselor/IBCLC Role Delineation” tool outlines the distinction between peer counselor and IBCLC scopes of services and provides a protocol for peer counselors to follow regarding when to consult with and/or refer a client to a BHP IBCLC. Examples of situations that require involving a BHP IBCLC include a client presenting with significant physiologic risk for low milk supply, nipple pain not alleviated by positioning and latch assistance, unresolved breast engorgement, mastitis and bacterial infections, or a condition that is contraindicated for breastfeeding. Peer counselors also involve a BHP IBCLC if infants are presenting with issues, such as signs of dehydration, diarrhea, poor weight gain or excessive weight loss, or an anatomic anomaly that may interfere with optimal breastfeeding like a cleft lip or palate.

 Peer counselors help bridge the gap in trust that can exist between clients from low-income minority backgrounds and the medical community and advocate for clients within the health care system. For example, peer counselors empower clients to express their breastfeeding goals to health care providers and help ensure clients’ wishes are respected. Peer counselors also conduct active outreach to clients via phone, email, text message, mail, and home visits to minimize loss-to-follow-up because clients could not be reached. Follow-up is important in this population because challenges that they experience, such as housing instability, often make it difficult for them to keep BHP appointments and respond to outreach attempts consistently.

The Hispanic Health Council offers a broad array of services complementary to BHP and sustains a network of many other health and social services available in the community. While peer counselors themselves do not directly address social determinants of health experienced by clients that may negatively impact breastfeeding, peer counselors inform the BHP program manager of these issues as they arise following a protocol. The manager then determines the services most appropriate for addressing the identified needs and links clients to these services. Examples of common social determinants of health that present challenges to clients include food insecurity, the need for affordable housing, payment of utilities, and lack of health insurance resulting in medical debt.

In-depth interviews and program materials offered particularly rich information on specific elements of the program, and therefore, the following sections focus on these elements.

#### Reclaiming breastfeeding as a cultural tradition

The first peer counselors engaged in the program named it “Lactancia Herencia y Orgullo” (Breastfeeding Heritage and Pride) to promote the concept of reclaiming breastfeeding as a cultural tradition and a feeling of pride rather than shame when practicing breastfeeding. This slogan underlies all aspects of the program and builds on the historical and cultural aspects of breastfeeding while empowering communities to practice it.

#### Hiring, training, and retaining peer counselors to serve in community and clinical settings

The Hispanic Health Council hires and trains women who have previously breastfed successfully for at least six months to serve as peer counselors. Many peer counselors join BHP without high levels of formal education or past work experience but are screened for their ability to handle the demands of the job, including the number of required work hours, ability to use a data management system for monitoring and evaluation, ability to learn and share evidence-based breastfeeding information to clients, and a non-judgmental attitude towards the population served by the program.

Peer counselors are trained in three phases. During the first phase, they participate in forty hours of classroom training guided by an evidence-informed training manual designed to equip them with lactation management and communication skills, as well as an ability to work effectively as part of a clinical team within health care facilities. The training adheres to the principles and techniques of popular education, also known as empowerment or Freirian education, in honor of popular educator, Paulo Freire [35]. Accordingly, the training values the experiential knowledge of peer counselors and considers that peer counselors themselves may have experienced a long history of economic, political, and cultural oppression. It also validates the life experiences and knowledge of peer counselors, promotes reflection and analysis of health problems and related social and economic issues, and supports the development of relevant solutions by peer counselors. In acknowledging that infant feeding practices are the product of a complex interplay of social, economic, political, health, and cultural factors, the training strongly fosters peer counselors’ respect and empathy for clients. At the end of the training, peer counselors must pass a written examination to assess their knowledge.

In the second phase of training, peer counselors shadow experienced peer counselors and the program IBCLC to gain hands-on, practice-based experience providing breastfeeding education and support and exposure to all aspects of BHP services. The IBCLC observes peer counselors, uses a skills assessment tool to assess their ability to perform their role, and confers with a program manager to determine when peer counselors are ready to deliver services independently. The final phase involves ongoing training; the IBCLC provides regular individual onsite guidance and supportive supervision and continued training for each peer counselor and conducts monthly training sessions for all peer counselors on timely topics, such as findings from recently published journal articles and breastfeeding challenges clients are facing. Peer counselors attend conferences and other relevant meetings to further enhance their knowledge and receive continuing education.

Peer counselors have opportunities to advance within the program. Several peer counselors have received their Certified Lactation Counselor (CLC) certification, and some are pursuing IBCLC certification, with one peer counselor becoming one of the first Puerto Rican IBCLCs in the US. All peer counselors receive a salary and health benefits, which is important for recruiting highly capable peer counselors, retaining them to ensure program sustainability, and promoting social justice:

*“We’ve also found, for social justice reasons and equity reasons, they [peer counselors] need salaries…it goes with social justice and what you’re doing in the community around people doing work that is valuable, that they need to get paid for.” (BHP designer and implementer)*

#### Role modeling and providing social support for breastfeeding

Peer counselors often have similar cultural roots and life experiences as their clients, making them uniquely suited to build trust as peers, serve as empathetic role models, provide social support, and share strategies for overcoming breastfeeding difficulties:

*“She [peer counselor] asked me questions about, how my nursing was going. She didn’t really—she wasn’t more like, “Oh, do this. Do that. Do this.” It was more like how am I doing? Do I need anything? She was more so on the end of… being a friend and then…it was, I guess, it would be like it was—it wasn’t more like a—she had medical insight, but it wasn’t like a doctor telling you, “Take this and take this.” It was…very comforting.” (BHP client)*

*“[Important for clients] to know that, hey, there’s somebody out there that I can reach out to when I’m in need. There are people there who want me to succeed. People like me, not somebody who is going to maybe look down upon me. But somebody who has gone through some, if not all, of one’s struggles. They’ve been successful. And therefore, sharing their experience with them and not just dictating what they want them to do.” (BHP designer)*

Further, peer counselors help prepare clients for addressing structural barriers to breastfeeding they may face, such as lack of support for breastfeeding in early care and education settings, limited or no maternity leave, and no time or spaces for breastfeeding at work.

#### Providing respectful, timely, and accessible education and counseling

By design, a central feature of BHP is that services are provided in a way that respects clients’ infant feeding preferences and choices. For example, many women view breastfeeding and formula feeding as an equal choice that they have the right to make but they are not familiar with public health recommendations for breastfeeding and benefits of breastfeeding for themselves and their infants. Therefore, peer counseling begins with peer counselors gently encouraging breastfeeding as the preferred practice by describing its benefits and ultimately respecting women’s infant feeding decisions:

*“I do remember, very clearly, a mom that was a very young prenatal Puerto Rican woman that we ask her, “Are you planning to breastfeed?” and then her question—okay, she looked at us and said, “Should I?” Like, no one had ever spoken with her, like her prenatal, antenatal care…So to begin with, it was a whole issue of bringing [the breastfeeding] option. It was done in a very respectful way, respecting their choices.” (BHP designer)*

BHP is also designed to provide clients and their families with timely breastfeeding peer counseling. In addition to protocols specifying key times to conduct in-person visits and phone contacts, peer counselors are widely available to answer clients’ questions and assist them in resolving breastfeeding challenges as they arise:

*“And if nobody else is there, people give it at the [snaps fingers]—on the spot. One, ’cause they feel so the other piece of all this, because of embarrassments, is lack of self-efficacy, right? So, they’re gonna try it and not know what to do to get rid of any of this and just say, “Forget this. It hurts.” So, we wanted people to fill the gap as role models in places and at moments when they could best help somebody continue as support and with technical capacity to help them get through the times that were difficult.” (BHP designer)*

BHP reduces access barriers to breastfeeding support by providing services, as well as devices to facilitate breastfeeding such as breast pumps, at no cost to clients. In addition, because peer counselors are based in clinics and hospitals, they can provide support to clients when they come in for prenatal care, are inpatient immediately following childbirth, or return to hospitals for follow-up visits with their health care providers. Home visits further promote access to breastfeeding support, since they eliminate clients needing to travel and arrange transportation to clinics and hospitals:

*“It was really convenient that these [counseling visits] were in the home. Because a lot of these moms…didn’t have a car. And even if you did…, if this is your first baby, …you’re still doing so much adjusting to the sleep deprivation of that first week or, like, I don’t expect any mom would’ve really wanted to get home and then come back to the hospital in 24 hours for [a] breastfeeding visit. If we showed up at her house, it was so convenient for her, because she’s right there …, she might’ve had to pack up kids and a diaper bag, and catch a bus to get to us… So that would’ve been a huge hassle.” (BHP designer)*

Home visits also enable peer counselors to learn about the level of support for breastfeeding a client has in her home environment, and to reach other members of the family who can support the client in meeting her breastfeeding goals:

*“You come into their house, then, –it…let you kinda see what other resources and help was around, too. …So that if you go into the home, and there’s a grandmother there then teaching the grandmother and mom simultaneously…or if dad’s around, dad can be shown how he can be helpful…how he can… take care of mom, get her the pillows to make this comfortable, what he can be doing to help…. And it just kind of reinforcing his role. So, …if there is anybody else in the house, they [peer counselors] tied them into the visit and… drew them in.” (BHP designer)*

#### Continuous quality improvement

BHP has a robust monitoring and evaluation system to assess process outcomes (e.g., recruitment rates, number of counseling visits), clients’ breastfeeding goals, and their breastfeeding outcomes such as any or exclusive breastfeeding. Data are collected by peer counselors during each client contact and entered into a data management system. Peer counselors use data from individual clients to tailor services to each client’s needs. In addition, data are aggregated and reviewed monthly to facilitate caseload and services management by peer counselors, quality assurance, and supervision by the program manager. BHP also uses a Program Impact Pathway analysis (PIP), a dynamic approach used to evaluate maternal-child program implementation by developing a causal map of the steps and processes linking program goals, activities, and outcomes and assessing critical quality control points along the pathway [36–38]. A PIP was co-developed by Yale School of Public Health and the Hispanic Health Council to frame the bi-annual quality improvement meetings with key stakeholders, including program leaders and managers, IBCLCs, peer counselors, and health care staff and providers from Trinity Health Of New England hospitals. Stakeholders discuss how well BHP is being implemented, identify bottlenecks, and reach consensus on ways to improve program delivery to ensure high-quality breastfeeding support for all clients.

### Program impact

Evidence from two randomized controlled trials has demonstrated that BHP improves breastfeeding outcomes among low-income minority populations in the US. In an initial trial conducted in 2004, Chapman et al. evaluated the effectiveness of the program by comparing breastfeeding outcomes between BHP clients (intervention group) and women in the control group [19]. The intervention group received breastfeeding support through one prenatal home visit, daily perinatal visits, 3 postpartum home visits, and phone contact as needed. Results showed that the percentage of women not initiating breastfeeding was lower in the intervention group than the control group (9 % vs. 23 %; relative risk, 0.39 [95 % CI: 0.18–0.86]). The percentage of women stopping breastfeeding was lower in the intervention group at both 1 month (36 % vs. 49 %; relative risk: 0.72 [95 % CI: 0.50–1.05]) and 3 months (56 % vs. 71 %; relative risk: 0.78 [95 % CI: 0.61-1.00]). Since this peer counseling model did not have any effect on exclusive breastfeeding rates, Anderson et al. tested a more intensive model in 2005 in a subsequent trial [33]. The intervention group received exclusive breastfeeding peer counseling support including three prenatal home visits, daily perinatal visits, nine postpartum home visits, and phone counseling as needed. At hospital discharge, a higher proportion of women in the control group compared with the intervention group had not initiated breastfeeding (24 % vs. 9 %) and were not exclusively breastfeeding (56 % vs. 41 %). At 3 months, 97 % of women in the control group compared with 73 % in the intervention group had not exclusively breastfeed during the previous 24 hours (relative risk: 1.33 [95 % CI: 1.14–1.56]). Similarly, women in the control group were more likely than those in the intervention group to not exclusively breastfeed throughout the first 3 months (99 % vs. 79 %; relative risk: 1.24 [95 % CI: 1.09–1.41]).

To identify those most responsive to BHP, both Chapman et al. and Anderson et al. conducted differential benefit response analyses using data from their respective trials [31, 32]. Chapman et al. found that multiparous women and those without clear prenatal breastfeeding intentions were more likely to initiate breastfeeding in response to BHP compared with their counterparts [31]. Women exposed to BHP who were partially breastfeeding on day one postpartum were more likely to breastfeed through 3 months than were women in the control group, indicating that peer counseling may reduce the negative effects of early formula introduction on breastfeeding duration [31]. Anderson et al. found that although all sub-groups benefited from BHP, non-Puerto Rican women (i.e., Hispanic women from countries such as Mexico, Peru, Dominican Republic, Colombia, Ecuador, and Honduras and Black women) responded better to BHP than Puerto Rican women [32]. Given that the peer counselors delivering counseling in this study were Puerto Rican, the findings suggest that ethnic minority women will respond to breastfeeding peer counseling interventions delivered by counselors from other ethnic minority groups [32].

## Discussion

BHP is one of the few evidence-based breastfeeding peer counseling programs available for low-income minority women in the US who often lack access to lactation services. BHP fills major gaps in breastfeeding support for this population by providing high-quality breastfeeding peer counseling in clinical and community settings. BHP clients receive breastfeeding counseling from rigorously trained and well supported peer counselors who are responsive to the specific cultural, contextual, and socio-economic needs of clients. When BHP clients experience breastfeeding challenges that necessitate more specialized knowledge and clinical expertise, they have access to support from a BHP IBCLC. It is reassuring that BHP fully adheres to the breastfeeding guidance from the World Health Organization published in 2018 [39]. Specifically, the program provides face-to-face counseling, begins counseling prenatally, and works with clients through one year postpartum. The program also provides counseling in clients’ homes and in clinical settings to optimize clients’ access to breastfeeding education and support when they need it and where it can be delivered effectively.

The experience and positive impact of BHP offers five key lessons that may be useful to consider when designing and implementing breastfeeding peer counseling programs:

- Community-engaged formative research and continuous community input: The BHP approach of co-designing programming and ensuring the fit of the intervention with the community’s needs and contexts may improve community engagement, acceptability of programming, and clinical outcomes [40].
- Training and retention of breastfeeding peer counselors: BHP has built a highly capable, sustainable workforce of community health workers. This has likely been possible because the Hispanic Health Council hires peer counselors who are committed to improving breastfeeding outcomes in their communities, successfully recruits new peer counselors as needed, provides peer counselors with rigorous training and supportive supervision, and offers them pay and benefits.
- Peer counselor and IBCLC scopes of services: Peer counselors receive supportive supervision from a BHP IBCLC and are empowered to support clients in preventing and overcoming a myriad of breastfeeding challenges. When an issue arises that is outside of peer counselors’ scope of services, a more specialized, higher-paid BHP IBCLC provides direct support to clients. This model enables BHP to provide high-quality support, while delivering support in a way that is cost-efficient.
- Continuum of breastfeeding support: BHP provides clients with breastfeeding support from pregnancy through one year postpartum. In addition, BHP delivers services in both clinical and community settings. Impact evaluations of BHP have demonstrated that this continuum of breastfeeding support across time and settings can improve breastfeeding outcomes among women who have some of the lowest breastfeeding rates in the US and may face more pronounced or additional challenges to achieving their breastfeeding goals than other subpopulations [19, 33, 41].
- Community-clinical partnerships: BHP can provide services in communities and clinics because of strong, equitable partnerships between the Hispanic Health Council and health care organizations, illustrating how essential it is that partnerships between community-based organizations and health care organizations are established to provide a continuum of breastfeeding support across settings.
- Protocol for linking clients with other health and social services: Connecting clients with services that can help address social determinants of health following protocols, as BHP does, is crucial for promoting equity in breastfeeding and health when serving low-income populations.
- Monitoring and evaluation: BHP’s sound monitoring and evaluation system supports continuous quality improvement. Benefits of engaging program leaders and managers, IBCLCs, peer counselors, and health care staff and providers in monitoring and evaluation activities include: strengthening community-clinical partnerships; ensuring that quality improvement efforts take into account diverse views, needs, and interests; fostering support for quality improvement efforts from all key stakeholders; and enhancing coordination of these efforts.

A strength of this case study was that we used the CEB Framework, which emphasizes cultural-contextual influences on high risk of obesity in racial/ethnic minority populations. By using multiple sources of information, we were able to triangulate findings and develop a rich description of BHP and the role it plays in promoting equity in breastfeeding. We captured the perspectives and opinions of multiple stakeholders by interviewing BHP designers and implementers, peer counselors, and clients. The case study also had some notable limitations. We did not capture perspectives of health care providers who work closely with peer counselors to coordinate and provide breastfeeding support for clients. Future work should include their perspectives on the value of integrating a community-based peer counseling program into clinical settings. To date, BHP has relied on grant funding since it was established nearly three decades ago. To support the scale-up and sustainability of the program, it is crucial that BHP services be reimbursable through public and private insurance. Research to evaluate the cost-effectiveness of BHP is needed to generate evidence for informing health care system decision makers and payers interested in investing in evidence-based programs to address inequities in breastfeeding. It will be important for payers to value investment in breastfeeding peer counseling as a prevention strategy that will yield long-term health benefits and reduce health care costs [1, 2, 4, 5, 42]. Furthermore, research to understand the extent to which social networks potentially amplify the effects of BHP by creating spillover effects to social contacts not directly served by the program (for example, breastfeeding knowledge and behaviors spreading from BHP clients to their friends and family) would be useful for informing decisions to implement and fund BHP [43]. If studies document social-network related spillover effects, these effects can be taken into account when assessing the reach and cost-effectiveness of BHP.

As emphasized by the CEB Framework, family and household level interventions such as BHP should be complemented by interventions at the community level. Examples of such interventions include the Baby-Friendly Hospital Initiative which promotes maternity care practices supportive of breastfeeding, maternity leave policies, and policies and programs to support breastfeeding in workplace and early care and education settings. These interventions should be co-designed with communities to ensure that services are contextually and culturally appropriate and meet the needs and wants of communities.

## Conclusions

Despite marked expansion of breastfeeding support for women in the US in recent years, large inequities in access to these services remain. BHP is an evidence-based example of a breastfeeding peer counseling model that improves equity in breastfeeding support by reaching women who would otherwise receive limited or no support to meet their breastfeeding goals.

## Data Availability

BHP materials may be available upon request. Qualitative data generated and analyzed during the study are not publicly available to maintain confidentiality but may be available from the corresponding author on reasonable request. All requests for materials and data should be made to the corresponding author at elizabeth.rhodes@yale.edu. Program impact evaluations have been published elsewhere and are available online.

## Abbreviations

BHP: Breastfeeding Heritage and Pride program
CEB Framework: Community Energy Balance Framework
CLC: Certified Lactation Counselor
IBCLC: International Board Certified Lactation Consultant
PIP: Program Impact Pathway analysis
US: United States
WIC: Special Supplemental Nutrition Program for Women, Infants, and Children
